# Dental Conscious Sedation for the Treatment of Children With Autism Spectrum Disorder: A Narrative Review

**DOI:** 10.7759/cureus.64834

**Published:** 2024-07-18

**Authors:** Abdulaziz Alyahyawi, Mohammed Barry, Narmin M Helal

**Affiliations:** 1 Pediatric Dentistry, Jazan Specialist Dental Hospital, Jazan Health Cluster, Jazan, SAU; 2 Pediatric Dentistry, King Abdulaziz University, Jeddah, SAU

**Keywords:** procedural sedation and analgesia, procedure sedation, : autism spectrum disorder, autism, conscious sedation

## Abstract

Conscious sedation has been shown to be a reliable behavior management tool that can be used during dental treatments in children who are less cooperative with dental treatment. The purpose of this study is to review the currently available research on the use of conscious sedation during dental procedures for children diagnosed with autism spectrum disorder (ASD). A web-based search for published articles was conducted. Different electronic databases were searched, including PubMed, Google Scholar, Online Review, and the Cochrane Library database, for papers published until February 2023. Studies providing descriptive protocols for dental conscious sedation for children with ASD were included. The search strategy found two studies that met the included criteria. The use of nitrous oxide in conjunction with oral benzodiazepines was found to be effective in sedating children with ASD. This review highlights the lack of research on sedation techniques for children with ASD. Future studies are needed to determine the specific types of sedative medications, their combinations and dosages, and the best methods for conscious sedation during dental procedures for autistic children.

## Introduction and background

Autism spectrum disorder (ASD) is a chronic neurodevelopmental condition marked by qualitative impairments in social interactions and communication patterns [1]. Patients with ASD typically have a small, repetitive, stereotyped range of interests [2]. Self-harming behavior, anger, temper tantrums, and mental symptoms are all behavioral problems seen in ASD patients.

There is still no established explanation for the causes of ASD. Researchers work to identify the cause, which, for many people, appears to be tied to a number of different circumstances. Also, it is thought that the etiology of autism lies in unidentified anomalies in a particular region of the brain. Several core regions have been suggested to mediate clinical signs of ASD, such as the frontotemporal lobe, frontoparietal cortex, amygdala, hippocampus, basal ganglia, and anterior cingulate cortex [3,4]. There are genetic and congenital diseases that have been linked to autism, such as oculocutaneous albinism, hearing impairment, muscular dystrophies, tuberous sclerosis, and phenylketonuria. Moreover, there have been reports of associations with a number of syndromes, including Moebius, Cornelia de Lange, Down, Goldenhar, Laurence-Moon Biedl, Noonan, Rett, and Laurence-Moon Biedl syndromes. Additionally, nongenetic prenatal disorders such as chemical exposure during pregnancy, mumps, rubella, toxoplasmosis, syphilis, and varicella infections have all been connected to autism. Additionally, recent studies have related maternal stress to the development of ASD in children [5]. Mercury is a potential contributing factor to autism, according to one theory. Many newborns have a hereditary propensity to retain heavy metals (copper, lead, aluminum, mercury, etc.) rather than having the body excrete them. These metals can accumulate in the brain, stay undetected, and induce serious neuronal degeneration for about six months after exposure [6]. As a result, it is believed that there is unquestionable data that environmental circumstances, strong genetic influences, and biological causes contribute to autism [7].

Typically, the Leiter International Performance Scale (LIPS), which assesses mental maturity and IQ, and the Diagnostic Checklist for Behavior Disturbed Children - E2, developed by Rimland in 1964 to evaluate children under the age of five, are used to make the diagnosis of autism. The Diagnostic Checklist for Behavior Disturbed Children - E2 consists of an 80-item questionnaire that parents must complete [8].

Dental procedures may be difficult for patients with ASD to cooperate with. According to reports, their interactions with dentists and their capacity to follow instructions while at the appointment pose the biggest challenges [9]. The literature has emphasized the difficulty of managing autistic patients and the necessity of looking into the most effective means of giving these children proper dental care. To improve the patient’s psychological health and, as a result, their quality of life, experts believe that early diagnosis, basic therapies, knowledge of how to speak to autistic children specifically, and long-term follow-up are all essential [10,11].

In general, children with autism prefer soft and sweetened foods, and they tend to pouch food inside the mouth instead of swallowing due to poor tongue coordination, thereby increasing their susceptibility to caries [12]. Given the high frequency of dental caries and periodontal disease in this population, patients with autism undoubtedly have a special need for attention. Notwithstanding the considerable heterogeneity of the included articles, a recent meta-analysis underlined this exact point by demonstrating a difference in caries of 60% and periodontal disease of 69.4% in the studied pool [13]. According to a recent review, children with ASD are more likely to experience oral traumas due to their hyperactivity, stereotyping, and self-harming behaviors [14]. They also have a higher risk of acquiring caries, periodontal disease, and changes in the oral flora. In order to promote patient compliance, identify potential lesions earlier, and intervene with adequate preventive and less invasive therapies, the authors once again emphasized the necessity for tailored approaches with early monitoring.

The biggest difficulty in providing dental care to children with ASD is their decreased capacity for social interaction and communication. Negative dental behavior could result from a variety of issues, including an inability to control emotions, repetitive bodily movements, hyperactivity, which may be linked to attention deficit disorder, and a low threshold for irritation [15]. Since these patients dislike even little changes in their environment and require continuity in their care, the dental team should be set up for atypical responses to sensory stimuli [16].

Due to the limitations faced while applying non-pharmacological behavior guidance techniques when treating children with ASD, conscious sedation has been shown to be a reliable behavior management tool that can be used during dental treatments in children who are less cooperative with dental treatment [17]. Although the morbidity and mortality risks connected with general anesthesia are significantly higher compared to those linked to conscious sedation, general anesthesia is typically administered to ASD patients for dental treatments [18]. The purpose of this study is to review the currently available research on the use of conscious sedation during dental procedures for children diagnosed with ASD.

## Review

A web-based search for published articles was conducted. Different electronic databases were searched, including PubMed, Google Scholar, Online Review, and the Cochrane Library database, for papers published up until February 2023. Studies providing descriptive protocols for dental conscious sedation for children with ASD were included. Studies were selected according to defined criteria, which are the following:

Study design and participants

All study designs for children with a diagnosis of ASD were considered. Studies examining human subjects ≤18 years old who received dental treatment under conscious sedation were included.

Intervention

Any protocol of conscious sedation, including inhalation, intranasal, intraoral, intravenous, or a combination of all, was considered.

Outcomes

The completion of dental treatment under conscious sedation for children with ASD was reported as the primary outcome measurement. The onset and duration of the sedative agent used and the evaluation of behavior during treatment were also reported as outcome measurements.

Results and discussion

Inhalation Sedation

The use of nitrous oxide as a behavior management technique for children with autism has been described in the literature [19]. In a retrospective study, Mangione et al. described the use of nitrous oxide inhalation sedation in combination with oral premedication for the dental treatment of 118 individuals with ASD. The use of nitrous oxide was carried out in 31% of cases with nitrous oxide inhalation sedation alone and in 46% with nitrous oxide inhalation sedation in conjunction with oral premedication [9]. The delivery of nitrous oxide necessitates a certain degree of communication with the patient, which might be challenging for patients with ASD [11,20]. Desensitizing techniques along with the use of nitrous oxide sedation were suggested as a combination by Watanabe. In his study, this approach was effective in 87.5% of the patients (average age, 11 years) who had previously had treatments but did not require physical constraints to undergo dental care [21]. In another study, the use of nitrous oxide was observed to be successful in ASD patients who present without severe behavioral problems and are able to cooperate; however, the number of patients who reported receiving the treatment was low [22]. Furthermore, in a recent retrospective analysis of dental treatment provided for 54 children using advanced behavior management techniques, it was found that 31% of children with ASD received successful dental treatment with inhalation sedation utilizing nitrous oxide/oxygen sedation. Additionally, 46% required oral premedication of midazolam or diazepam with nitrous oxide/oxygen sedation in order to achieve successful outcomes [9].

It is worth mentioning that nitrous oxide inhibits the enzyme methylenetetrahydrofolate reductase (MTHFR) substantially, which can severely limit DNA synthesis and result in megaloblastic alterations in blood cells and bone marrow. Inhibition of DNA synthesis and rapid MTHFR synthesis typically cause no problems in neurotypically healthy individuals [23]. Nevertheless, there are case reports detailing the deaths of children who had a certain genetic enzyme impairment related to these chemical pathways [23,24]. The MTHFR enzyme is also essential for the metabolism of folate. Children who lack vitamin B12 are also more likely to suffer morbidity or mortality. Moreover, it was reported that these children underwent prolonged procedures while under general anesthesia in the operating room [24]. Regardless, nothing in the literature indicates that the use of nitrous is discouraged in children with ASD. However, some autistic children are reported to have biochemical anomalies in the metabolism of folic acid, vitamin B12 deficiency, and MTHFR malfunction [25,26]. Therefore, dentists must be aware of the causes of the parents’ concerns regarding the use of nitrous oxide and be prepared to assist them in making informed decisions. The risk of nitrous oxide use is minimal, if not nonexistent [27]. Yet, if the patient’s parents are strongly opposed to the use of nitrous oxide, the dentist may choose to utilize a different sedation technique, have the patient undergo further pre-anesthetic testing for gene deficiency, or speak with the patient’s medical practitioners.

Oral, Intranasal, and Intravenous Sedation

Generally, if appropriate basic nitrous/oxide-oxygen sedation techniques are ineffective in delivering reliable outcomes for patients with ASD, the use of parenteral sedation may be utilized as a necessary alternative to enable routine dental work. Sedation in this patient group is acceptable with no additional medical risk over that seen in the general population because there are few medical issues related to autism that are related to sedation. This approach has been advised by several authors in the past [28-30]. Although nitrous oxide, chloral hydrate, hydroxyzine, and diazepam are routinely used in pediatric dentistry, their effectiveness varies due to their varied administration methods, dosages, and frequencies [31]. Braff and Nealon discussed the effective pharmacological interventions for the dental care of patients diagnosed with ASD. They concluded that several sedative medications may be useful, and a pharmacological combination might help achieve a proper level of sedation. In their trial, diazepam and chloral hydrate or hydroxyzine together had a good sedative effect on autistic patients [32].

This observation was supported by Lowe and Jedrychowski, who suggested and encouraged the use of a diazepam and hydroxyzine combination with nitrous oxide/oxygen and reported acceptable results [33].

Fukuta et al. examined the sedative effects of intranasal midazolam at a dosage of 0.2 mg/kg when combined with nitrous oxide/oxygen inhalation during dental treatment for children with disabilities, including those with ASD. They showed that midazolam had a productive sedative effect at the start of dental treatment. The technique they described in this study was practical and had no major adverse events [34]. Moreover, oral midazolam was found to be an effective premedication for patients with signs of autism who required general anesthesia for medical interventions [35].

Midazolam and diazepam are both sedative-hypnotic medications in the benzodiazepine family group. Diazepam is widely accepted as a sedative agent in pediatric dentistry because it is most frequently used as an anxiolytic with a high margin of safety and few negative effects. The maximum single dose of oral diazepam is 10 mg, and the suggested dosage ranges from 0.15 to 0.5 mg/kg. Oral administration produces an onset in 30 to 45 minutes, and its duration of action is four to six hours [36]. Moreover, some research indicates that diazepam has a sedative effect on children lacking cooperation and children with disabilities [37]. On the other hand, it has been demonstrated that midazolam, a medication that is frequently used to sedate patients prior to anesthesia in both adults and children [38], consistently provides appropriate safety and effective behavior management. Peak plasma levels of oral midazolam are obtained within 30 minutes, and its duration of action ranges from 45 to 60 minutes. Pediatric patients should take oral midazolam in doses of 0.25 to 1 mg/kg, with a maximum of 20 mg per dose. It is quickly absorbed by the gastrointestinal tract. Midazolam has a surprisingly effective sedative impact, especially given the minimal and unremarkable side effects observed [36].

In a study that was conducted to evaluate how effective oral diazepam and midazolam were in the sedation of autistic patients undergoing dental treatment, in a cross-over design trial with 13 participants aged 5.8 to 14.7 years, the treatment protocol included nitrous oxide/oxygen inhalation in addition to oral administration of either diazepam 0.3 mg/kg or midazolam 0.5 mg/kg. It was shown that both medications were effective and successful agents for the sedation of children with ASD. However, it was observed that midazolam was more successful at controlling sleep, activity, and crying behavior, and it gave the patient a homogeneous reaction. Yet, the duration of action was shorter than that of diazepam. Despite having a longer duration of action, diazepam was less effective and caused the patient’s response to vary more. Therefore, it was concluded that, although both drugs were effective sedative agents, midazolam was observed to be more effective in regulating patients’ behavior at times of increased stimulation such as injections, extractions, etc. [17]. A similar response could be seen in healthy children [39].

Generally, due to the lack of research in the literature that specifically looked at pediatric patients with a specific diagnosis of ASD receiving dental care, we were able to investigate a smaller number of studies. In some studies, pediatric patients were evaluated within heterogeneous groups, but the articles failed to provide sub-analyses of the findings that would have allowed us to extrapolate the data regarding pediatric autistic patients. Additionally, conscious sedation was frequently exclusively employed as a premedication intervention prior to general anesthesia, but our goal was to assess if conscious sedation used for dental procedures had any implications for the routine clinical management of young patients with ASD. Even in light of the difficulties that can arise from dental procedures done while a patient is under general anesthesia, it is crucial to understand more about conscious sedation for autistic children. In a study that was published in 2021, it was found that ASD patients had a higher incidence of negative behavioral effects than healthy individuals eight hours after surgery, including difficulty walking and nausea [40]. The challenges that are faced when providing dental care for children with ASD lead to the necessity for further research on the subject of conscious sedation, as it is widely known that children with ASD have issues getting dental care due to a lack of cooperation abilities, which reduces their chances of receiving effective dental therapy [41].

## Conclusions

Both inhalation and parenteral sedation techniques could be used for the dental management of children with ASD. Midazolam was found to be more effective in lowering body movement and crying behaviors compared to diazepam. Future studies are needed to determine the specific types of sedative medications, their combinations and dosages, and the best methods for conscious sedation during dental procedures for autistic children. Future research should also examine the effectiveness of conscious sedation in dental procedures for patients with ASD.

## Appendices

**Table 1:** Characteristics of the included studies ASD, autism spectrum disorder

Author and country	Design	Number of participants	Age (years)	Severity of ASD	Sedation protocol	Dental treatment	Success of sedation
Pisalchaiyong et al. (2005) [17], Thailand	Prospective randomized clinical trial	13	5.8-14.7	Not mentioned	Nitrous oxide inhalation in combination with oral administration of diazepam 0.3 mg/kg or midazolam 0.5 mg/kg	Restorations; endodontic; restorative periodontic treatment; primary teeth extractions; sealant and fluoride applications	Midazolam was more effective than diazepam as the duration of stimulation increased; midazolam was more effective in lowering movement and crying behaviors.
Mangione et al. (2020) [9], Italy	Retrospective cohort	83	4-17	Not mentioned	Oral premedication, nitrous oxide/oxygen inhalation sedation, and nitrous oxide/oxygen inhalation sedation + oral premedication	Sealants; fluoride varnish applications; supragingival scaling and/or subgingival root planning; radiographic; tooth restorations (direct fillings without pulp involvement); endodontic treatments (pulpotomies and root canal treatments) and extractions	13% of children received treatment with oral premedication, 31% with nitrous oxide inhalation sedation alone, and 46% with nitrous oxide inhalation sedation + oral premedication.

## References

[1] Yesilyurt C, Sezer U, Ayar MK, Alp CK, Tasdemir T (2013). The effect of a new calcium-based agent, Pro-Argin, on the microhardness of bleached enamel surface. Aust Dent J.

[2] Prakash S, Pai VK, Dhar M, Kumar AA (2016). Premedication in an autistic, combative child: challenges and nuances. Saudi J Anaesth.

[3] Frith U (1997). The neurocognitive basis of autism. Trends Cogn Sci.

[4] Brambilla P, Hardan AY, di Nemi SU, Caverzasi E, Soares JC, Perez J, Barale F (2004). The functional neuroanatomy of autism. Funct Neurol.

[5] Alamoudi RA, Al-Jabri BA, Alsulami MA, Sabbagh HJ (2023). Prenatal maternal stress and the severity of autism spectrum disorder: a cross-sectional study. Dev Psychobiol.

[6] Kern JK, Geier DA, Sykes LK, Haley BE, Geier MR (2016). The relationship between mercury and autism: a comprehensive review and discussion. J Trace Elem Med Biol.

[7] Amaral DG (2017). Examining the causes of autism. Cerebrum.

[8] Baird G, Cass H, Slonims V (2003). Diagnosis of autism. BMJ.

[9] Mangione F, Bdeoui F, Monnier-Da Costa A, Dursun E (2020). Autistic patients: a retrospective study on their dental needs and the behavioural approach. Clin Oral Investig.

[10] Herrera-Moncada M, Campos-Lara P, Hernández-Cabanillas JC, Bermeo-Escalona JR, Pozos-Guillén A, Pozos-Guillén F, Garrocho-Rangel JA (2019). Autism and paediatric dentistry: a scoping review. Oral Health Prev Dent.

[11] Aljubour AA, AbdElBaki M, El Meligy O, Al Jabri B, Sabbagh H (2024). Culturally adapted dental visual aids effect on behavior management during dental visits in children with autism spectrum disorder. J Contemp Dent Pract.

[12] Zerman N, Zotti F, Chirumbolo S, Zangani A, Mauro G, Zoccante L (2022). Insights on dental care management and prevention in children with autism spectrum disorder (ASD). What is new?. Front Oral Health.

[13] da Silva SN, Gimenez T, Souza RC (2017). Oral health status of children and young adults with autism spectrum disorders: systematic review and meta-analysis. Int J Paediatr Dent.

[14] Ferrazzano GF, Salerno C, Bravaccio C, Ingenito A, Sangianantoni G, Cantile T (2020). Autism spectrum disorders and oral health status: review of the literature. Eur J Paediatr Dent.

[15] Kamen S, Skier J (1985). Dental management of the autistic child. Spec Care Dentist.

[16] Lowe O, Lindemann R (1985). Assessment of the autistic patient's dental needs and ability to undergo dental examination. ASDC J Dent Child.

[17] Pisalchaiyong T, Trairatvorakul C, Jirakijja J (2005). Comparison of the effectiveness of oral diazepam and midazolam for the sedation of autistic patients during dental treatment. Pediatr Dent.

[18] Soldani F, Manton S, Stirrups DR, Cumming C, Foley J (2010). A comparison of inhalation sedation agents in the management of children receiving dental treatment: a randomized, controlled, cross-over pilot trial. Int J Paediatr Dent.

[19] Zanelli ME, Volpato LE, Ortega AL, Borges AH, Aranha AM (2016). Nitrous oxide for dental treatment in patients with infantile autism: a literature review. Rev Sul-Brasil Odontol.

[20] Ritwik P, Gupta K (2020). Use of nitrous oxide in children with special health care needs. Clint Dent Rev.

[21] Watanabe T (1992). Efficacy of a combination of desensitization and nitrous oxide inhalation in sedating autistic patients during dental treatment. Pediatr Dent J.

[22] Loo CY, Graham RM, Hughes CV (2009). Behaviour guidance in dental treatment of patients with autism spectrum disorder. Int J Paediatr Dent.

[23] Selzer RR, Rosenblatt DS, Laxova R, Hogan K (2003). Adverse effect of nitrous oxide in a child with 5,10-methylenetetrahydrofolate reductase deficiency. N Engl J Med.

[24] Nagele P, Zeugswetter B, Wiener C, Burger H, Hüpfl M, Mittlböck M, Födinger M (2008). Influence of methylenetetrahydrofolate reductase gene polymorphisms on homocysteine concentrations after nitrous oxide anesthesia. Anesthesiology.

[25] James SJ, Melnyk S, Jernigan S (2006). Metabolic endophenotype and related genotypes are associated with oxidative stress in children with autism. Am J Med Genet B Neuropsychiatr Genet.

[26] Blaylock RL (2008). A possible central mechanism in autism spectrum disorders, part 1. Altern Ther Health Med.

[27] Malamed SF (2018). Inhalation sedation: complications. Sedation (Sixth Edition).

[28] Davila JM, Jensen OE (1988). Behavioral and pharmacological dental management of a patient with autism. Spec Care Dentist.

[29] Shapira J, Mann J, Tamari I, Mester R, Knobler H, Yoeli Y, Newbrun E (1989). Oral health status and dental needs of an autistic population of children and young adults. Spec Care Dentist.

[30] Klein U, Nowak AJ (1998). Autistic disorder: a review for the pediatric dentist. Pediatr Dent.

[31] Jo CW, Park CH, Lee JH, Kim JH (2017). Managing the behavior of a patient with autism by sedation via submucosal route during dental treatment. J Dent Anesth Pain Med.

[32] Braff MH, Nealon L (1979). Sedation of the autistic patient for dental procedures. ASDC J Dent Child.

[33] Lowe O, Jedrychowski JR (1987). A sedation technique for autistic patients who require dental treatment. Spec Care Den.

[34] Fukuta O, Braham RL, Yanase H (1993). The sedative effect of intranasal midazolam administration in the dental treatment of patients with mental disabilities. Part 1. The effect of a 0.2 mg/kg dose. J Clin Pediatr Dent.

[35] van der Walt JH, Moran C (2001). An audit of perioperative management of autistic children. Paediatr Anaesth.

[36] Malamed SF (2017). Oral sedation. Sedation (Sixth Edition).

[37] Diner MH, Fortin RC, Marcoux P, Legault V (1988). Behavioral influences of rectal diazepam in solution on dental patients with mentally and physically handicapping conditions. Spec Care Dentist.

[38] Feld LH, Negus JB, White PF (1990). Oral midazolam preanesthetic medication in pediatric outpatients. Anesthesiology.

[39] Dionne R (1999). Oral midazolam syrup: a safer alternative for pediatric sedation. Compend Contin Educ Dent.

[40] Tran J, Chen JW, Trapp L, McCormack L (2021). An investigation of the long and short term behavioral effects of general anesthesia on pediatric dental patients with autism. Front Oral Health.

[41] Logrieco MG, Ciuffreda GN, Sinjari B (2021). What happens at a dental surgery when the patient is a child with autism spectrum disorder? An Italian study. J Autism Dev Disord.

